# Bedtime Routines Intervention for Children (BRIC) project: results from a non-randomised feasibility, proof-of concept study

**DOI:** 10.1186/s40814-022-01039-7

**Published:** 2022-04-06

**Authors:** George Kitsaras, Iain A. Pretty, Julia Allan

**Affiliations:** 1 Dental Health Unit, The University of Manchester, Manchester, UK; 2 Institute of Applied Health Sciences, University of Aberdeen, Aberdeen, UK

**Keywords:** Child, Wellbeing, Sleep, Parenting, Behaviour change

## Abstract

**Background:**

Bedtime routines are highly recurrent family activities with implications for children’s wellbeing, development and health.

**Aims:**

The objective of this study is to co-develop and test in a feasibility, proof-of-concept study a bedtime routines intervention using text messages aimed at first-time parents with young children.

**Methods:**

Fifty first-time parents with children aged 1–3 years were recruited for this study. Parents received a text message-based intervention for 7-consecutive nights which provided support and information on achieving optimal bedtime routines. Parents completed pre- and post-intervention questionnaires focusing on children’s sleep, bedtime routines and parental mood disturbance. Feedback was provided at the end of the study.

**Results:**

Recruitment target and high retention with 98%, or 49 out of 50 participants completing the study were achieved. Pre- and post-intervention, there were improvements in total children’s sleep with children sleeping longer and having less disrupted sleep overall (MD = − 7.77 (SD = 17.91), *t*(48) = − 3.03, *p* = .004, CI (− 12.91, − 2.63) and in overall quality of bedtime routines (MD = − 5.00, SD = 7.01, *t*(48) = − 4.98, *p* < .001, CI (− 7.01, − 2.98). Parental mood disturbance decreased pre- to post-intervention (MD *=* 5.87, SD = 15.4*3*, *t*(48) = 2.66), *p* = .010, CI (1.44, 10.30). Parents provided positive feedback about the intervention and valued the support that was provided to them.

**Conclusions:**

Bedtime routines were successfully altered with short-term benefits for children’s sleep and parental mood. Future research will need to utilize a more robust, longitudinal approach for a definite exploration of sustained changes in bedtime routines and their long-term implications for children and parents.

**Supplementary Information:**

The online version contains supplementary material available at 10.1186/s40814-022-01039-7.

## Key messages regarding feasibility

- What uncertainties existed regarding the feasibility?
  - Can we recruit and retain sufficient number of first-time parents into a text message-based intervention for bedtime routines?
  - Will a text message-based intervention result in initial, changes on key child sleep, parental mood and bedtime routine quality changes?
  - Will first-time parents with young children accept a text message-based intervention, find it practical and affordable?
- What are the key feasibility findings?
  - Sufficient number of participants were recruited with a 98% retention rate during the study.
  - Preliminary, changes were found pre- and post-intervention on children’s sleep quality, bedtime routines quality and parental mood disturbance.
  - Parents provided positive feedback regarding the intervention.
- What are the implications of the feasibility findings for the design of the main study?
  - Minor changes to the intervention following feedback should take place before progressing to a larger study.
  - A longitudinal study will be necessary to assess long-term changes in bedtime routines sustained over-time.
  - Randomisation and inclusion of more metrics will be vital to showcase robust changes in children’s wellbeing and development as well as parental mental health and family functioning.

## Background

Family life revolves around behavioural routines [1]. From morning to mealtimes to bedtime each family develops and implements their own routines and rituals that both complement their schedules and accomplish important tasks for the day [1]. Those routines might differ among families with some key similarities with regards to the behaviours that take place during these routines and rituals [2]. All family routines are essentially a series of behaviours [3], typically centred around particular time points or common activities.

From the wide range of routines and rituals within family life, bedtime routines have particular importance for children, parent(s) and/or caregivers alike. Bedtime routines can be described as a series of activities that take place in the hour prior to bed [2, 4]. Optimal bedtime routines require a range of recurrent, adaptive and interactive behaviours to be consistently carried out by parents and children [2, 5]; ideally including toothbrushing, avoidance of electronic devices including TV, avoidance of snacks and drinks other than water or unflavoured milk, reading of a book or storytelling between the parent and the child, as well as other activities that promote positive interactions between parents and children [2, 5]. Available research has highlighted the wide-ranging importance of bedtime routine behaviours for children’s wellbeing, development and health as well as for parental mood and wellbeing. Children with optimal routines tend to sleep better at night [6], have fewer problems at school the next day [5], score higher on tests of cognitive functioning and school readiness [5], have less dental decay and dental disease [4, 7] and achieve better overall scores on key well-being, development and health measurements [5]. As for parents, those in households with optimal bedtime routines for children report less stress, higher parental competence and confidence as well as more positive family functioning and even higher marriage satisfaction [4, 8, 9]. Bedtime routines are not a silver bullet that can address all developmental, health and well-being issues for children and families. Nevertheless, there is an ever-increasing body of literature that highlights their broad importance and relevance to better physical, mental and social outcomes for children and parents.

Given the importance of bedtime routines, it is vital to identify what methods and forms of support may be most effective for parents and/or caregivers to establish optimal routines. This is likely to be particularly important for first time parents who have never had the need to develop and implement a bedtime routine for children before [10]. Previous research with first time parents has highlighted the importance of focusing resources and support on this particular group. Research from the Family Nurse Practitioners program in the US and in the UK for example, has shown how important it is for first time parents in particular, to ‘learn something right’ when they first learn it [11]. Previous studies have reported successfully improving bedtime routines; however, most of these studies focused on time consuming and resource intensive interventions that might be challenging to implement in routine practice (and within publicly funded social care systems), or on designs that informed parents what to do without providing support to change and sustain behaviours overtime. Also, many bedtime routine interventions failed to engage in co-design with users or in-depth exploration of the target behaviours involved prior to intervention development and implementation.

With bedtime routines comprising a series of behaviours, it is pertinent to undertake a robust, evidence-based approach to intervention development using established behaviour change frameworks like the Behavioural Change Wheel (BCW) [12]. Following the principles outlined in the BCW framework, it is possible to ensure that the target behaviour and the barriers and facilitators for that behaviour are clearly conceptualised before moving on to intervention development and implementation. The same principles highlight the criteria likely to influence the feasibility and success of a behavioural intervention through the APEASE criteria (Affordability, Practicability, Effectiveness, Accessibility, Safety and Equity) and the need for feasibility and proof-of-concept studies prior to larger, more robust and long-term evaluations [13].

Given the intrinsic role that bedtime routines play in family life, it is also important to use co-design approaches to ensure the developed intervention will work for the intended population in real life. Design principles, like design thinking, advocate for an agile, user-inclusive and collaborative approach for the development of interventions providing a basis for building more effective and successful interventions [14]. Finally, with regards to delivering interventions, cost-effective, user-friendly approaches should be considered first. One cost-effective, easy to use and empirically supported approach is to use text messages [15]. Text messages have been used extensively within health behaviour change interventions; however, thus far, such interventions have never been used to deliver a standalone intervention for bedtime. Also, text messages avoid the need for internet connectivity and smartphone technology resulting in more equitable access with ownership of working mobile phones at an all-time high across the UK.

### Objectives

The present feasibility, proof-of-concept study aims to test a theory-informed text message-based intervention for achieving optimal bedtime routines for first-time parents with young children. Specifically, the study (a) examines recruitment and retention rates during the study with an aim to recruit 50 participants within a specified recruitment period and retain at least 90% of them by the end of the study, (b) explores preliminary changes in three key outcomes (children’s quality and duration of sleep, bedtime routine quality and parental mood) pre- and post-intervention and (c) gathers participant feedback on intervention acceptability and suggestions for moving forward with additional research projects guided by user-involvement and input.

## Methods

### Overview

A detailed protocol for this study has been published previously [10]. This study forms part of a wider Bedtime Routines Intervention for Children (BRIC) project involving specific work packages over an 18-month period. Despite lack of randomisation, the CONSORT statement extension to pilot and feasibility studies [16] has been used to present the results of this study omitting non-applicable items from the checklist [17]. Additional file A presents the completed CONSORT checklist.

### Intervention

An iterative approach was used throughout the wider BRIC project, guided by behavioural principles for designing, developing and implementing interventions. Two main work packages were included in the wider, BRIC project. Work package 1 focused mainly on getting a better understanding of common barriers and facilitators establishing and implementing optimal bedtime routines [3]. Table 1 summarises the key barriers identified during this process. Once key barriers and facilitators had been identified, appropriate behaviour change techniques (BCTs) were selected and translated into a text message-based intervention for first-time parents. Table 1 presents an overview of how appropriate BCTs were selected to address the key barriers. Barriers were mapped into the theoretical domains framework (TDF) domains (a framework which summarises 84 possible determinants of behaviour into overarching theoretical domains allowing for a comprehensive exploration of all possible determinants of bedtime routines) [12] which were then mapped into the relevant COM-B (Capability, Opportunity, Motivation–Behaviour) components resulting in the identification of appropriate intervention functions and BCTs.

**Table 1:** Process for selecting appropriate BCTs to address key barriers identified in work package 1 of the BRIC project which were then translated and used in the intervention

Barrier	TDF domain and structure	COM-B component	Intervention function
Feeling tired at the start of the routine especially during the week	Memory, Attention & Decision process + Cognitive overload Emotion + Burn-out & Negative affect	Capability (psychological) Motivation (automatic)	Training, education & enablement Persuasion & incentivization
Lack of consistent and reliable information, knowledge and support especially when first having bedtime routines	Knowledge + Procedural knowledge Skills & Skills development, Practice & Skills assessment	Capability (psychological)	Training, education & enablement
Routines are habitual and difficult to change	Behavioural regulation + Self-monitoring, breaking habit & action planning Beliefs about capabilities + Perceived behavioural control, perceived competence, self-esteem & self-efficacy	Capability (psychological) Motivation (reflective)	Training, education & enablement Persuasion & incentivization

For the identified intervention functions, the following behaviour change techniques are applicable to change and sustain changes to the target behaviour.

- Education: (a) information about consequences (proximal/distal), (b) prompts and (c) cues
- Persuasion: (a) credible source
- Incentivization: (a) self-monitoring of behaviour
- Training: (a) instruction on how to perform the behaviour
- Enablement: (a) goal setting, (b) review behaviour/goals

Once appropriate BCTs were selected, they were used to develop the content of the intervention text messages. A small group of first-time parents (*N* = 7), who were recruited during work package 1, provided feedback on different versions of the intervention text messages. In total, 2 development cycles were initiated with parents testing each design and providing feedback that was then used to make necessary changes. Feedback was provided through online sessions with the parents. Engaging parents in the design process was important to ensure that the content struck the right tone for parents with young children, the content was easy to comprehend and follow, the length of each text message was appropriate and finally, sources used in the text messages were perceived as reliable from parents. The final intervention was a series of text messages that would be sent to parents each night for 7 consecutive nights and timed to arrive approximately 1 h before their child’s normal bedtime. Each text message was unique and included content designed around a relevant BCT with the aim of increasing engagement in optimal bedtime routine behaviours.

Through this process and the feedback provided by parents, the decision was made to provide participants on the feasibility, proof-of-concept study with two alternative versions of the intervention to choose between each night: a detailed version and a shorter, summary version. Regardless of the version chosen, participants received text messages for 7 consecutive nights. The detailed version included lengthier, more detailed information, practical support and messages aimed at motivating parents in order to achieve an optimal bedtime routine. In the detailed version, a total of 10 text messages were sent to participants each night. The detailed version lasted a total of 5 min each night. The summary version covered the same content but more succinctly and acted more as a brief reminder of the key behaviours to be performed each night. In the summary version, a total of 6 text messages were sent to participants each night. The summary version lasted a total of 3 min each night.

At the start of the intervention week, parents were asked (by text message) if they were ready to receive the intervention. At that stage and before receiving the intervention, parents were offered the option to delay the text messages, defer them to the next day or drop out of the study all together. Text messages were personalized with the participant’s first name and participants decided the time they wished to receive the text messages. Text messages included a series of links to external, evidence-based and credible resources and information from the National Health Service (NHS) and Public Health England (PHE) in the UK.

### Feasibility, proof-of-concept study

In order to test the feasibility of the developed intervention, *n* = 50 first time parents with one child aged 1–3 years were recruited into a 7-day study where they received the intervention by text message on a nightly basis.

#### Recruitment

Due to disruptions caused by COVID-19, all recruitment was undertaken online. The research team joined a series of parent groups on social media where posts informing potential participants about the study were shared with prior permission from group moderators/administrators. Also, a series of nurseries and kindergartens across England were invited to share information about the study with parents. Exclusion criteria included (a) inability to speak English, (b) not having a working mobile phone, (c) having more than 1 child and (d) having children under the age of 1 or over the age of 3. Parents had to make first contact with the research team in response to a study advertisement, and on doing so were provided with a more detailed participant information sheet and an online consent form. Parents were offered up to 48 h to decide if they wanted to participate in the study. Those who agreed to take part in the study were assigned unique participant IDs. Recruitment took place between September 2020 and January 2021.

#### Procedure

Each participant completed a brief demographics form with questions on age, gender, child’s age and gender, ethnicity, employment and level of education. Also, each participant completed a contact form where they provided their mobile phone number and indicated the time and day on which they wish to start receiving the intervention. Before receiving the intervention, each participant completed three online questionnaires to assess the three main outcomes of children’s sleep quality and duration (Child Sleep Habits Questionnaire), parental mood (Profile of Mood States) and bedtime routine quality (Bedtime Routines Questionnaire). The text message intervention lasted for 7 consecutive nights and text messages arrived at the time indicated by participants in the contact forms (and in general, 1 h before the child was due to be in bed). Each night, participants had the option to delay, defer or fully opt-out from the study. At the end of the 7 days, the three questionnaires were completed again online. Feedback was requested from each participant at the end of the study and was provided by both anonymized text surveys and online-hosted focus groups. Once a participant completed all steps of the study, they received a £10 voucher (to be used online) that was sent via post to their home address.

#### Measures

The Children’s Sleep Habit Questionnaire (CSHQ) [18] contains 22 questions answered on a 5-point Likert scale (always, usually, sometimes, rarely and never) and produces 4 subscales relating to children’s sleeping habits: bedtime routine activities, sleep-related behaviours, night waking and morning wake up behaviours. Higher scores indicate better quality sleep. The CSHQ has been used extensively within sleep research with high outcome validity and reliability.

The original version of the Bedtime Routines Questionnaire (BRQ) [19] contains 31 questions on a 5-point Likert scale (almost never, occasionally, half the time, often and nearly always) spread across four target areas: weekdays, weekends, how upset the child gets if he or she does not perform some activities and a list of 15 bedtime-related activities). For the purpose of this study, an adaptive version of the BRQ was used where the 15 bedtime related activities were condensed to 8 overarching activities that better reflected the activities targeted by the intervention, for example, in the original BRQ the separate activities of ‘hug/kiss caregiver’, ‘say goodnight to family members’, ‘get tucked in’, ‘put on pajamas’, ‘cuddle’ and ‘say prayers’ were grouped together as ‘interactive, positive activities between parent/child’ with specific examples offered to participants to further explain the term. Higher scores indicate more optimal bedtime routines. The validity and reliability of the BRQ has been indicated in work by Henderson and Jordan [19].

Finally, as a longstanding, validated, reliable and easy to complete measurement, the Abbreviated version of the Profile of Mood States (POMS) [20] has been selected in order to assess parental mood disturbance. POMS contains 40 statements that participants need to score using a 5-point Likert scale (not at all, a little, moderately, quite a lot and extremely). Seven subscales are calculated from scores: parental tension, anger, fatigue, depression, self-esteem, vigor and confusion. Total negative scores (tension, anger, fatigue, confusion and depression) are added together and then the positive scores are subtracted subtracting the total positive scores (self-esteem and vigor) to produce the total mood disturbance score where higher scores indicate higher parental mood disturbance (i.e. greater negative mood).

#### Feedback

At the end of the intervention, parents were sent an anonymized feedback text survey where they were asked a series of open and close-ended questions regarding their participation in the study, the quality, frequency and content of the text messages as well as space for offering recommendations on what needs to change. Parents were also invited to an optional focus group held online to further discuss their participation in the study and provide more comments, feedback and suggestions on how to improve the intervention moving forward.

#### Analyses

Descriptive data analyses were used for demographic information as well as for the feedback provided by participants through text surveys. Differences in pre- and post-intervention scores were compared using paired samples t-tests for each of the three outcomes used in the study. Exploratory sub-scale analyses with pre and post scores and t-test results were also conducted with corrections (Bonferroni correction) for multiple analyses. For feedback provided by participants, thematic analyses were performed on the focus group data at the end of the study to identify common themes arising during the discussions. Descriptive data and frequency counts were analysed for feedback provided via anonymized text surveys. Cost was calculated based on the cost of a single text message send to a UK-registered number.

## Results

### Sample characteristics

In total, 50 first-time parents were recruited for the study. Table 2 provides an overview of sample characteristics including age and gender of parents taking part in the study, their educational and employment status and details on ethnicity. Index of Multiple Deprivation (IMD), a national instrument for calculating and quantifying deprivation across England, deciles were calculated based on participants’ postcodes. Low deciles indicate higher deprivation.

**Table 2:** Sample characteristics

Characteristic		*N* (% of total sample)
Mean Age (parents) (in years)	M = 34.1 (SD = 4.3)	
Gender (parents)	Male	5 (10%)
	Female	45 (90%)
Mean Age (child) (in months)	M = 25.1 (SD = 9.5)	
Gender (child)	Male	26 (52%)
	Female	24 (48%)
Ethnicity	White ethnic groups	35 (70%)
	Asian, British-Asian	9 (18%)
	Black, British-Black, Caribbean	4 (8%)
	Multiple ethnic groups	2 (4%)
Employment	Full-time employed	18 (36%)
	Part-time employed	21 (42%)
	Stay-at-home parent	7 (14%)
	Self-employed	3 (6%)
	Student	1 (2%)
Education	University (undergraduate or higher)	17 (34%)
	High-school and post-high school	33 (66%)
IMD deciles	1 (most deprived)	8 (16%)
	2	6 (12%)
	3	5 (10%)
	4	5 (10%)
	5	5 (10%)
	6	4 (8%)
	7	3 (6%)
	8	7 (14%)
	9	2 (4%)
	10 (least deprived)	5 (10%)

### Recruitment and retention rates

During the recruitment period, 75 potential participants approached the research team with 50 deciding to participate in the study and complete necessary forms including a consent form resulting in a 67% recruitment rate. During the study, there was only one participant who dropped out after completing the pre-study questionnaires. All remaining participants (*N* = 49) completed all measurements resulting in a high retention rate of 98%.

### Type of intervention

Each night, participants were presented with two versions of the intervention (detailed and summary). The first night, all participants opted in for the detailed version. For the second night, most participants (30/50) opted for the summary version. Thereafter, for nights 3 to 7, most participants (40/50) opted for the summary version. Only a small number of participants (3/50) interchanged between the different versions, with no specific pattern, beyond the third night. In the analyses presented below, there were no changes depending on the type of intervention used by participants in the study.

### Effects on children’s sleep, quality of bedtime routines and parental mood disturbance

**Fig. 1:**
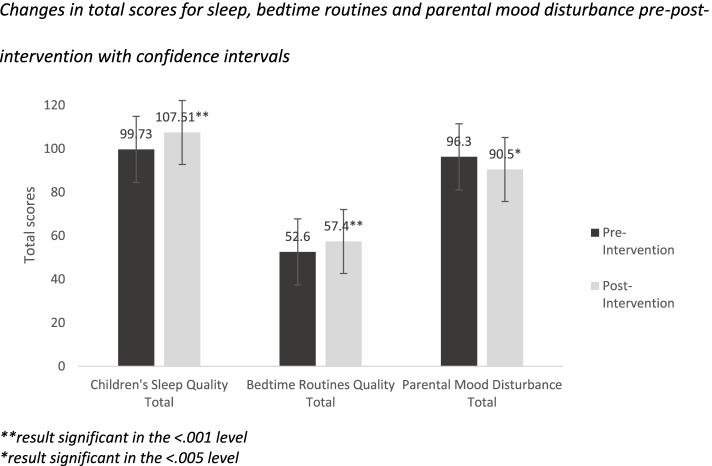
Changes in total scores for sleep, bedtime routines and parental mood disturbance pre-post-intervention with confidence intervals. **result significant in the < .001 level. *result significant in the < .005 level

Paired sample *t* tests were performed to explore changes in pre- and post-intervention scores on the three measurements used in the study: children’s sleep, bedtime routines and parental mood disturbance. For total scores across all three questionnaires, there were positive changes pre- and post-intervention: children’s sleep quality improved pre-post-intervention (MD = − 7.77 (SD = 17.91), *t*(48) = − 3.03, *p* = .004, CI (− 12.91, − 2.63)). Also, overall bedtime routine quality improved pre-post (MD = − 5.00, SD = 7.01, *t*(48) = − 4.98, *p* < .001, CI (− 7.01, − 2.98)). Finally, total parental mood disturbance decreased from pre-post-intervention (MD *=* 5.87, SD = 15.43, *t*(48) = 2.66), *p* = .010, CI (1.44, 10.30)). Figure 1 presents a visualization of total score changes pre-post-intervention.

Changes were also observed on specific subscales within each of the three outcome measures used in this study. More specifically, for children’s sleep, there were improvements across three out of its four subscales: (a) bedtime routine behaviours were improved pre-post-intervention (MD = − 3.51, SD = 9.47, *t*(48) = − 2.59, *p* = .013, CI (− 6.23, − 0.78), (b) night waking episodes improved pre-post-intervention (MD = − 1.67, SD = 3.74, *t*(48) = − 2.61, *p* = .050, CI = − 2.75, 0.79)) and (c) morning wake up behaviours were also improved pre-post-intervention (MD = − 1.81, SD = 3.50,*t*(48) = − 3.62, *p* = .001, CI (− 2.82, − 0.80)). For children’s sleep, the only subscale that failed to show changes pre-post-intervention were the sleep-related behaviours (i.e. naps during the day, restlessness during sleep, grinding teeth and snoring while asleep) (MD = − 1.46, SD = 5.48, *t*(48) = − 1.87, *p* = .067, CI (− 3.04, 0.10)).

Six out of the seven subscales in the parental mood disturbance measure also showed improvements pre- and post-intervention. On reported negative emotions, parents reported feeling less tense pre-post-intervention (MD = 1.14, SD = 3.54,, *t*(48) = 2.25, *p* = .028, CI (.12, 2.06), less angry pre-post-intervention (MD = 0.75, SD = 2.28, *t*(48) = 2.31, *p* = .025, CI (0.10, 1.41) and less fatigued post-intervention (MD = 1.24, SD = 4.53, *t*(48) = 2.10, *p* = .050, CI (− .05, 2.54); and less confused post-intervention (MD = 0.57, SD = 1.99, *t*(48) = 2.01, *p* = .050, CI (− 0.04, 1,14)). With regards to positive emotions, parents reported both higher self-esteem post-intervention (MD = − 1.10, SD = 2.83, *t*(48) = − 2.72, *p* = .009, CI (− 1.91, − 0.28)) and higher vigor post-intervention (MD = − 0.81, SD = 2.86, *t*(48) = − 2.00, *p* = .05, CI = − 1.64, 0.08).

Finally, for the bedtime routines questionnaire, all three of its subscales indicated improvements pre- and post-intervention. Bedtime routines improved both at weekdays pre-post-intervention (MD = − 0.87, SD = 1.95, *t*(48) = − 3.21, *p* = .002, CI (− 7.01, − 2.98)) and at weekends pre-post intervention (MD = − 1.32, SD = 2.22, *t*(48) = − 4.16, *p* < .001, CI (− 1.96, − 0.68)). Finally, bedtime routine-related activities were improved pre- (MD = − 2.97, SD = 4.79, *t*(48) = − 4.52, *p* < .001, CI (− 4.17, − 1.41)). Bedtime routine activities covered a total of eight targeted areas. Within those eight areas, six produced important results pre- and post-intervention. Parents read more to their children pre-post-intervention (MD = − 0.71, SD = 1.24, *t*(48) = − 4.027, *p* < .000, CI (− 1.00, − 0.35)); parents also interacted more with their children pre-post intervention (MD = − 0.63, SD = 1.09, *t*(48) = − 4.05, *p* < .000, CI (− 0.94, − 0.31)); children watched less TV the hour before bed pre-post-intervention (MD = 1.00, SD = 1.44, *t*(48) = 4.85, *p* < .001, CI (0.58, 1.41)); children also played less with electronic devices the hour before bed pre-post-intervention (MD = 0.36, SD = 0.88, *t*(48) = 2.91, *p* = .005, CI (0.11, 0.62)); children were also allowed fewer snacks and drinks other than water and unflavoured milk the hour before bed pre-post-intervention (MD = 0.57, SD = 1.33, *t*(48) = 2.98, *p* = .004, CI (0.18, 0.95)) and finally, children brushed their teeth more pre-post-intervention (MD = 0.61, SD = 1.77, *t*(48) = − 2.41, *p* = .020, CI (− 1.12, − 0.10)). The only two bedtime routine activities that failed to show changes pre-post-intervention were children’s play *t*(48) = − .43, *p* = 668 and having a shower or a bath before bed *t*(48) = − .86, *p* = .392, but neither of these behaviours were explicitly targeted in the current intervention. Table 3 presents an overview of percentage change for all bedtime routine activities.

**Table 3:** Changes in bedtime routine activities pre- and post-intervention with percentage changes reflecting how much more or less frequently parents gave responses

Reading before bed	N	Pre-intervention (%)	Post-intervention (%)	Change (%)
Almost never	49	4	0	− 4
Occasionally	49	14	0	− 14
Half of the time	49	10	2	− 8
Often	49	8	12	+ 4
Nearly always	49	64	84	+ 20
Parent-child interactions	49	Pre-intervention (%)	Post-intervention (%)	Change
Almost never	49	8	0	− 8
Occasionally	49	18	0	− 18
Half of the time	49	0	0	0
Often	49	14	12	− 2
Nearly always	49	60	86	+ 26
Watching TV before bed	49	Pre-intervention (%)	Post-intervention (%)	Change
Almost never	49	38	64	+ 26
Occasionally	49	16	16	0
Half of the time	49	8	10	+ 2
Often	49	20	6	− 14
Nearly always	49	18	2	− 16
Playing with electronic devices before bed	49	Pre-intervention (%)	Post-intervention (%)	Change
Almost never	49	80	96	+ 16
Occasionally	49	12	2	− 10
Half of the time	49	0	0	0
Often	49	6	0	− 6
Nearly always	49	2	0	− 2
Having snacks and/or drinks before bed (excl. water and/or unflavoured milk)	49	Pre-intervention (%)	Post-intervention (%)	Change
Almost never	49	60	84	+ 24
Occasionally	49	20	10	− 10
Half of the time	49	4	0	− 4
Often	49	6	2	− 4
Nearly always	49	10	2	− 8
Brushing teeth before bed	49	Pre-intervention (%)	Post-intervention (%)	Change
Almost never	49	16	4	− 12
Occasionally	49	8	1	− 7
Half of the time	49	4	4	0
Often	49	2	8	+ 6
Nearly always	49	70	80	+ 10

This table summarised percentage changes on the frequency of six bedtime routine activities that showed changes pre- and post-intervention. Two activities (play and shower/bath before bed) are not included since they did not show changes

### Feedback and cost

Feedback was provided through anonymous text message surveys at the end of the project and through online focus groups. A total of 47/50 participants provided anonymized feedback via text surveys while 25 participants joined an online focus group. Participants overall reported a very positive experience awarding an average satisfaction score of 9 out of 10 for the intervention. Participants also commented positively on the use of and interaction with the text messages they received, scoring a 9.5 out of 10 for ease of use. Participants reported high acceptability of the intervention with an average score of 9 out of 10 as well as high practicability with an average score of 8.5 out of 10. There were no reported safety issues from participants and only 1 out of 47 participants mentioned cost as a factor that might needs to be considered in the future. Also, 43 participants (88% of all participants) said they will recommend this intervention to family and friends if it was more widely available. Finally, participants expressed no outright preference with regards to the two versions of the intervention with both versions (detailed and summary) receiving equally positive reviews and feedback.

The focus group discussed participants’ experiences more broadly while also exploring what should change in the intervention and what should remain moving forward. In terms of areas that might need to be changed in the future, participants wanted to see (a) a more dynamic and personalized intervention that allowed participants to choose the level of support, the frequency of text messages and the content of text messages, (b) some participants expressed a desire for a richer interface through the use of multimedia content along the lines of a mobile application or electronic resources alongside the text messages and finally and (c) parents would have liked to see both earlier and later ages included in the intervention (before the age of 1 and past the age of 3). In terms of things parents wanted to keep in any future iterations of the intervention, they strongly advocated for (a) maintaining the friendly, fact-based and concise nature of advice offered to them and (b) the easiness of the intervention in terms of interacting with it every night.

Each participant received on average 7 text messages per night resulting in a total of 49 text messages during the 7 nights of the study. Each text message costs on average £0.04 to send and £0.007 to receive. Therefore, for each participant, there was a cost of £0.04 × 49 = £1.96 for sending text messages over the 7-night period. Also, each participant was therefore charged a total of £0.007 × 49 = £0.34 for receiving the text messages over the 7-night period. That figure (£0.34) does not account for unlimited text messaging that is usually offered to individuals with a monthly mobile phone contract in the UK.

## Discussion

This feasibility, proof-of-concept study implemented and tested a theory and user informed, text message-based intervention for achieving optimal bedtime routines for first-time parents. Sufficient numbers of parents were recruited into the study and the retention rate was high. Preliminary data indicate beneficial effects across three key outcomes: children’s sleep quality, bedtime routine quality and parental mood disturbance pre- and post-intervention. At the end of the study, participants provided positive feedback and expressed their support and desire to see such an intervention more widely available.

### Recruitment and retention

The first objective of this feasibility, proof-of-concept study was to explore if we can successfully recruit *N* = 50 participants during a pre-specified recruitment period and retain at least 90% of participants till the end of the study including the completion of all study measurements. Despite adaptations to our previously published protocol [10] due to the COVID-19 pandemic which rendered our original recruitment plan obsolete, we were able to recruit our target sample within our pre-specified period. All recruitment took place online with electronic consent forms. An ethics amendment was essential however, no delays on the completion of the project occurred. Retention rate of 98% (*N* = 49) was achieved surpassing our initial target of 90%.

### Effects on children’s sleep

Based on the results of the study, there were positive changes pre-post-intervention with regards to children’s sleep quality as assessed by the Child Sleep Habits Questionnaire. Changes were also suggested across three out of the four subscales within the questionnaire. A wide body of available research has indicated a strong link between the quality of bedtime routines and children’s sleep, so this finding is in line with existing research in the area [2]. Sleep is a salient feature of overall health with poor sleep hygiene affecting children’s development, school performance, mood and cognitive functioning and development [21]. Also, children’s sleep is associated with parental sleep with the former affecting the latter resulting in poor sleeping habits for parents when children’s sleep is poor [2].

### Bedtime routines

Bedtime routine scores were improved pre-post-intervention indicating the potential improvement in the quality of routines experienced by parents and children as a result of the intervention. Bedtime routines improved not only on weekdays but also on weekends suggesting that the beneficial effects of the intervention were still apparent during periods where the evidence suggests that routines are often relaxed. Most bedtime routine activities (i.e. reading before bed, brushing teeth etc.) were improved pre- and post-intervention, with only two activities that were not improved (i.e. having a shower or a bath before bed and children’s play before bed). Both were behaviours not specifically targeted by the intervention. The pattern of effects—i.e. changes in targeted behaviours but not in associated but non-targeted behaviours—suggest that the intervention operated as intended.

For the activities where changes were observed, brushing is an important one to highlight. A quarter of parents reported not consistently brushing their children’s teeth at night before the intervention. Post-intervention, there was an increase in the number of parents who reported brushing their children’s teeth always or nearly always at night. Despite advancements in preventing dental disease, there are still pockets of poor dental health in the community and not brushing teeth at night, as well as sub optimal oral hygiene practises in the morning and throughout the day, can increase the likelihood of dental decay [22]. Dental decay, a mainly preventable disease, can have subsequent implications for children’s well-being and development with unnecessary dental pain when the disease progresses, missed hours and days at school with pain also affecting children’s sleep [4, 23, 24]. In some cases, children with advanced decay will need to undergo dental extractions under general anaesthetic resulting in additional negative consequences [25].

### Parental mood disturbance

Parental mood disturbance was assessed by the profile of mood states with changes pre- and post-intervention. Parents felt less tense, less fatigued, less confused, less angry post intervention while also reporting higher vigour and self-esteem. Reflecting on the common barriers to enacting an optimal bedtime routine that parents reported in a previous piece of work, fatigue as well as lack of motivation for change were two of the most common barriers identified [3]. Therefore, if the present intervention offers parents the tools, tips and information necessary to achieve optimal routines, the present results indicate that parents will feel less tired, less fatigued and more motivated to change their routines and may therefore be more able to sustain positive changes over time. Such effects are likely to be cyclical and self-reinforcing and may therefore contribute to the likelihood that positive changes will be sustained over time.

### Reflecting on the APEASE criteria

Considering the APEASE criteria (Affordability, Practicability, Effectiveness, Accessibility, Safety and Equity), the study results overall indicate that five criteria were satisfied. Affordability and equity were not mentioned as an issue from the majority of participants who provided feedback, and virtually all participants (from all socioeconomic backgrounds) were able to access and interact with the intervention text messages through existing mobile plans. Cost was kept to a minimum with £0.34 charged to participants’ mobile phones and only when they had pay-as-you-go contracts in place. Safety was not an issue in the current study as the intervention did not introduce any risk to participants. Participants reported the intervention, in its current format, to be both practical and acceptable with no problems fitting it around their bedtime schedules. Our high retention rate (98%) in part reflects the non-invasive and practical nature of the intervention in its current format. Finally, the preliminary data collected on the effects of the intervention on three key metrics of children’s sleep, bedtime routines and parental mood disturbance show positive indications with regards to its effectiveness.

### Strengths and limitations

#### Strengths

This study, and the BRIC project more widely, showcased a series of strengths. An evidence-based, stepped approach was taken from the beginning of the project to reflect established behavioural principles on designing, developing, implementing and evaluating interventions. That allowed for the exploration of barriers and facilitators as well as for user-inclusivity across the design and development of the intervention. The intervention itself was tested within a feasibility and proof of concept study with a diverse socioeconomic and sociodemographic sample. Standardised measures were used to quantify changes in key outcomes pre- and post-intervention. Also, there was a wide use of feedback mechanisms throughout the study resulting in a wide range of opportunities for learning and reflecting on what worked and what needs to change in the future. Finally, this study first devised in 2019, had to quickly adapt to the rapid changes created by a global pandemic with all its operations and procedures shifted online. Despite the unprecedented changes brought by COVID-19, this study managed to complete all its tasks and objectives within time and with no shortcomings, indicating the likely practicality and ease of delivering a similar intervention in practice.

#### Limitations

The main limitations of this study are the lack of a control group and the use of a convenience sample. A randomised control trial is required to examine pre- and post-intervention changes in outcomes; however, the present study was focused on determining whether the developed intervention using text messages for bedtime routines was feasible for use with first time parents. This stepped approach allowed the concept underpinning the intervention to be tested without the expense of a RCT design behind it. The convenience sampling approach may have limited the generalisability of findings, but the final sample was relatively diverse in terms of socioeconomic and demographic mix. Also, the requirement for participants to speak and understand English as this could have hindered the participation of families from marginalised groups. Moving forward, more language options for this type of intervention will be considered. The lack of total hours of sleep for children is another limitation as is the variation on the level of the intervention parents and children received each night given the choice for a detailed or summary version. Monitoring of engagement with different versions of the intervention will need to be in place in future work to better understand how it might affect results.

### Future directions

With a successful feasibility, proof of concept study complete, the immediate focus for the BRIC project will be a robust, larger scale randomised controlled trial with a longitudinal follow up to determine the effects (both short and long-term) of a text message-based intervention for bedtime routines with first-time parents and their children. The focus of this longitudinal, controlled study should be on a series of important outcomes linked to bedtime routines including children’s oral health, school readiness, socio-emotional development, cognitive functioning and sleep as well as on parents’ socioemotional wellbeing and family functioning more broadly. In addition, some changes may be made to the intervention itself on the basis of the detailed and constructive feedback provided by participants in the current study. Finally, alongside a robust controlled study, considerations should be made on how to best integrate any such intervention with routine practice for real world delivery to first-time parents.

## Conclusion

Bedtime routines encompass many behaviours, and optimal routines have clear health, development and wellbeing benefits for children and parents alike. Achieving and sustaining optimal bedtime routines has the potential to positively impact on a wide range of future outcomes for children while simultaneously offering parents some vital support and immediate benefits on a day-to-day basis. Cost-effective (a few pence per day), user-friendly interventions, like the present text message intervention for bedtime routines are feasible and have the potential to help parents achieve optimal routines. Additional work is necessary to conclusively establish the long-terms effects of such an intervention.

## Supplementary Information

**Additional file 1.** CONSORT checklist.

## Data Availability

The datasets used and/or analysed during the current study will be available from the corresponding author on reasonable request at the end of the study.

## Abbreviations

BCW: Behaviour Change Wheel
TDF: Theoretical Domains Framework
BCTs: Behaviour Change Techniques
APEASE criteria: Acceptability, Practicability, Effectiveness, Affordability, Safety and Equity criteria
CONSORT statement: Consolidated Standards of Reporting Trials
NHS: National Health Service (UK)
PHE: Public Health England
CSHQ: Children’s Sleep Habits Questionnaire
BRQ: Bedtime Routines Questionnaire
POMS: Profile of Mood States
